# Visualization and characterization of *Pseudomonas syringae* pv. tomato DC3000 pellicles

**DOI:** 10.1111/1751-7915.13385

**Published:** 2019-03-05

**Authors:** Gabriela A. Farias, Adela Olmedilla, María‐Trinidad Gallegos

**Affiliations:** ^1^ Department of Soil Microbiology and Symbiotic Systems Estación Experimental del Zaidín (EEZ‐CSIC) Granada Spain; ^2^ Department of Biochemistry, Cell and Molecular Biology of Plants Estación Experimental del Zaidín (EEZ‐CSIC) Granada Spain

## Abstract

Cellulose, whose production is controlled by c‐di‐GMP, is a commonly found exopolysaccharide in bacterial biofilms. *Pseudomonas syringae* pv. tomato (Pto) DC3000, a model organism for molecular studies of plant–pathogen interactions, carries the *wssABCDEFGHI* operon for the synthesis of acetylated cellulose. The high intracellular levels of the second messenger c‐di‐GMP induced by the overexpression of the heterologous diguanylate cyclase PleD stimulate cellulose production and enhance air–liquid biofilm (pellicle) formation. To characterize the mechanisms involved in Pto DC3000 pellicle formation, we studied this process using mutants lacking flagella, biosurfactant or different extracellular matrix components, and compared the pellicles produced in the absence and in the presence of PleD. We have discovered that neither alginate nor the biosurfactant syringafactin are needed for their formation, whereas cellulose and flagella are important but not essential. We have also observed that the high c‐di‐GMP levels conferred more cohesion to Pto cells within the pellicle and induced the formation of intracellular inclusion bodies and extracellular fibres and vesicles. Since the pellicles were very labile and this greatly hindered their handling and processing for microscopy, we have also developed new methods to collect and process them for scanning and transmission electron microscopy. These techniques open up new perspectives for the analysis of fragile biofilms in other bacterial strains.

## Introduction

Bacterial biofilms can be defined as sessile‐structured communities attached to a surface whose cells are embedded in a self‐produced polymeric matrix composed of proteins, extracellular DNA, lipids and different types of exopolysaccharides (Costerton *et al*., 1995). In addition to immobilizing bacteria and providing a mechanical stability to the biofilm, the matrix traps nutrients and various biologically active molecules, such as quorum sensing signals, and behaves as an external digestion system, since it also contains enzymes that degrade different components of the matrix, nutrients or other substrates, making the products available to cells which facilitates their absorption. In addition, this matrix functions as a shield protecting the bacterial community against predators, drying, UV rays, different antimicrobial agents and even the immune response of eukaryotic hosts (Sutherland, 2001; Donlan, 2002; Stoodley *et al*., 2002; Ramey *et al*., 2004; Van Houdt and Michiels, 2005; Flemming *et al*., 2016). Particularly, the formation of aggregates, microcolonies or biofilms on the plant leaves or roots allows bacteria to better adapt to that environment. Biofilm formation improves their chances of survival since it is a way of maintaining a bacterial critical mass in a specific location for a period of time sufficient to develop beneficial or antagonistic interactions with the host plants (Monier and Lindow, 2004; Danhorn and Fuqua, 2007).

The development of a biofilm takes place on almost any solid biotic or abiotic surface in a process in which microorganisms undergo profound changes during their transition from planktonic (free‐swimming) cells to the complex surface‐attached community. The process begins when free cells encounter a surface and adhere reversibly to it. Then, they divide and the daughter cells extend around the initial site of attachment forming a microcolony that is now irreversibly adhered to the surface. At a later stage, the bacterium secretes different compounds, such as exopolysaccharides, proteins, nucleic acids and lipids, which constitute the biofilm matrix, until the microcolonies are completely absorbed by it, giving rise to a mature biofilm. Finally, the process concludes (or re‐starts) with the release of some motile cells that will colonize new surfaces. Biofilm architectures are highly variable, ranging from open structures containing channels and columns of bacteria, to structures with no obvious pores and densely packed regions of cells (Costerton *et al*., 1995; Watnick and Kolter, 1999; Kuchma and O'Toole, 2000; O'Toole *et al*., 2000; Wimpenny *et al*., 2000; Hall‐Stoodley *et al*., 2004).

Although the canonical definition of biofilm includes its association with a solid surface and to date most attention has focused on biofilms formed from the colonization of solid–liquid (S‐L) or air–solid (A‐S) interfaces, there are multicellular structures similar to biofilms at the air–liquid (A‐L) interface. The colonization of the A‐L interface has been much less studied, but these biofilms, also called pellicles, can be selectively advantageous for aerobic or facultative aerobic bacteria since they provide access to both the gaseous (e.g. oxygen) and liquid (e.g. nutrient) phases (Davey and O'Toole, 2000; Koza *et al*., 2009; Nait Chabane *et al*., 2014). The requirements for the formation of this type of biofilm are also cell contacts, exopolysaccharide accumulation and, in addition, the ability to growth in static conditions (Friedman and Kolter, 2004; Ude *et al*., 2006; Robertson *et al*., 2013; Koza *et al*., 2017). A‐L biofilms form more complex structures and require a higher level of organization, compared with S‐L biofilms, owing to the lack of a solid surface on which the growth can be initiated (Branda *et al*., 2005). It has been shown that although polysaccharides such as alginate or levans can be present in these biofilms, cellulose is one of the main components of their extracellular matrix (Spiers *et al*., 2003; Ude *et al*., 2006; Pérez‐Mendoza *et al*., 2014).

*Pseudomonas syringae* pv. tomato (Pto) DC3000 causes bacterial speck on tomato and *Arabidopsis* and is a model organism for molecular studies of plant–pathogen interactions (Preston, 2000; Buell *et al*., 2003; Xin and He, 2013). Pto DC3000 enters the plant through wounds or leaf stomata and then replicates within the apoplast, causing chlorosis and necrotic lesions or programed cell death in incompatible interactions (Hirano and Upper, 2000; Xin and He, 2013). Bacterial motility facilitates the pathogen–host interactions and promotes colonization, penetration and invasion of host tissues. In Pto DC3000, the flagella master regulator FleQ is essential for its motility since it not only controls flagella production, but also the production of the biosurfactant syringafactin (Vargas *et al*., 2013; Nogales *et al*., 2015). This biosurfactant is essential for swarming motility but does not affect swimming motility (Berti *et al*., 2007; Nogales *et al*., 2015). Other key bacterial processes, like the stress response, quorum sensing, secondary metabolism and virulence, are under the regulation of the Gac‐rsm pathway (Chatterjee *et al*., 2003, 2007; Vargas *et al*., 2013). GacS/GacA is a two‐component system conserved in numerous Gram‐negative bacteria where GacS is a sensing histidine kinase that responds to environmental signals by activating the GacA response regulator by phosphorylation (Hrabak and Willis, 1992; Laville *et al*., 1992). GacA, in turn, activates the expression of the regulatory RNAs called rsm, which sequester the RsmA/CsrA proteins, preventing the post‐transcriptional regulation they exert on certain mRNAs (Valverde *et al*., 2003; Reimmann *et al*., 2005; Lapouge *et al*., 2008). The chain of command in the Gac‐rsm cascade of Pto DC3000 has not been identified conclusively but different studies suggest that Pto DC3000 has seven GacA‐controlled small RNAs (rsmX1‐5, rsmY, rsmZ) and five RsmA‐like RNA‐binding proteins (Heeb *et al*., 2006; Kulkarni *et al*., 2006; Lapouge *et al*., 2008; Moll *et al*., 2010; Vargas *et al*., 2013; Ferreiro *et al*., 2018).

The exopolysaccharides are also important for the interaction with the plant and Pto DC3000 likely produces an acetylated form of cellulose whose synthesis requires the *wssABCDEFGHI* operon (Ude *et al*., 2006; Pérez‐Mendoza *et al*., 2014; Prada‐Ramírez *et al*., 2016). This operon encodes proteins for the bacterial cellulose synthase (WssB and WssC), secretion of cellulose (WssA, WssD and WssE) and others that resemble those involved in alginate acetylation (WssG, WssH and WssI), and it is controlled at transcriptional level by AmrZ. In fact, AmrZ acts as a bifunctional regulator in Pto DC3000, repressing cellulose production and several proteins involved in the c‐di‐GMP metabolism, and activating motility, alginate production and virulence (Prada‐Ramírez *et al*., 2016).

Cyclic di‐GMP (c‐di‐GMP) is a second messenger used in signal transduction in a wide variety of bacteria. This ubiquitous messenger is a key factor in the regulation of the bacterial transition from a free and mobile lifestyle to a sessile and in association forming biofilms. To this end, it inhibits different forms of motility whilst it stimulates biofilm formation (Flemming *et al*., 2016; Valentini and Filloux, 2016; Jenal *et al*., 2017). And this applies to different types of biofilms, such as those formed at the S‐L, S‐A and A‐L surfaces, and rdar (red, dry and rough), WS (wrinkly spreader) and RSCV (rugose small‐colony variants) colonies (Rainey and Travisano, 1998; Branda *et al*., 2005; Römling, 2005; Yildiz and Visick, 2009; Armitano *et al*., 2014). In *P. aeruginosa*, the mucous colonies and the RSCV commonly isolated from patients with cystic fibrosis are associated with high levels of c‐di‐GMP and a great ability for biofilm formation (Meissner *et al*., 2007; Hay *et al*., 2009; Starkey *et al*., 2009; Malone *et al*., 2010). Previous work in our laboratory showed that artificial increase of intracellular c‐di‐GMP levels (overexpressing PleD*, a constitutive diguanylate cyclase from *Caulobacter crescentus*), stimulated the production of cellulose and alginate, enhanced pellicle formation under aerobic and static conditions and generated a colony phenotype similar to the rdar or WS (Pérez‐Mendoza *et al*., 2014). These pellicles easily precipitated, suggesting a strong adhesion among the cells, but a weak union to the abiotic surfaces, both of the borosilicate tubes and polystyrene multiwell plates (Robertson *et al*., 2013; Pérez‐Mendoza *et al*., 2014). The formation of pellicles at the A‐L interface is also observed in other strains of *Pseudomonas,* and in most of them, cellulose has been identified as the main component of the extracellular matrix (Spiers *et al*., 2003; Ude *et al*., 2006; Robertson *et al*., 2013; Armitano *et al*., 2014; Pérez‐Mendoza *et al*., 2014). However, a Pto DC3000 mutant unable to produce cellulose (*ΔwssBC*) also formed biofilms at the A‐L interface, albeit with a different appearance and consistency, in the presence of high c‐di‐GMP levels. The same phenotype has been observed in other strains of *Pseudomonas* lacking the cellulose synthesis operon, which indicates that high levels of c‐di‐GMP can activate the synthesis of other exopolysaccharides, such as alginate, levans, Pel or Psl (Friedman and Kolter, 2004; Laue *et al*., 2006; Ude *et al*., 2006; Lee *et al*., 2007; Starkey *et al*., 2009; Mann and Wozniak, 2012; Whitney and Howell, 2013). In fact, Pto DC3000 exhibited an increase in alginate production in the presence of PleD* (Pérez‐Mendoza *et al*., 2014).

Different imaging techniques have been proved useful to study the structure of biofilms including light microscopy, confocal scanning laser microscopy, atomic force microscopy and scanning or transmission electron microscopy (Franklin *et al*., 2015; Azeredo *et al*., 2017). However, the application of these techniques requires the adaptation of the different protocols to process the samples depending on the biofilm type and characteristics. Pellicles are very labile biofilms that are not easy to handle, which is a major problem to successfully complete the different stages of processing required for their study by microscopy. To analyse Pto DC3000 pellicles by scanning electron microscopy, we have developed a method using activated carbon cloths, and to study sections of these pellicles by light or transmission electron microscopy, we have introduced a pre‐embedding step in low melting agarose in the embedding procedure. These extra steps have allowed us the correct collection of these fragile biofilms, their processing and the acquisition of images using different microscopy techniques.

## Results

We previously showed that Pto DC3000 was able to form pellicles under aerobic and static conditions with the artificial increase of the intracellular c‐di‐GMP levels by the overexpression of the heterologous diguanylate cyclase PleD* (Pérez‐Mendoza *et al*., 2014). The colonization of the A‐L interface requires special mechanisms to prevent, on one hand, that bacteria sink by the gravity force and, on the other, their dispersion in the liquid media. These mechanisms involve flagella‐mediated motility, the production of biosurfactants or the synthesis of an extracellular matrix that prevents the biofilm from disintegrating in the liquid medium (Armitano *et al*., 2014). Biofilm flotation can also be favoured by its adhesion to the walls of the culture vessel (Robertson *et al*., 2013). To analyse these mechanisms, we have compared the formation of Pto DC3000 pellicles using different mutants, both in the absence and in the presence of PleD*. Mutants assayed were *fleQ* (which lacks the central regulator of both motility and biofilm traits in *Pseudomonas*), *syfA* (which does not produce the biosurfactant syringafactin), and three mutants unable to produce different exopolysaccharides: *wssBC* (which does not produce cellulose), *alg8* (which does not produce alginate) and *amrZ* (which does not produce alginate but overproduces cellulose). Finally, we also used the *gacA* mutant that lacks the global GacA response regulator controlling quorum sensing, response to stress, secondary metabolism and virulence (Chatterjee *et al*., 2003; Vargas *et al*., 2013; Pérez‐Mendoza *et al*., 2014; Prada‐Ramírez *et al*., 2016).

### Characterization of Pto DC3000 pellicles

**Figure 1:**
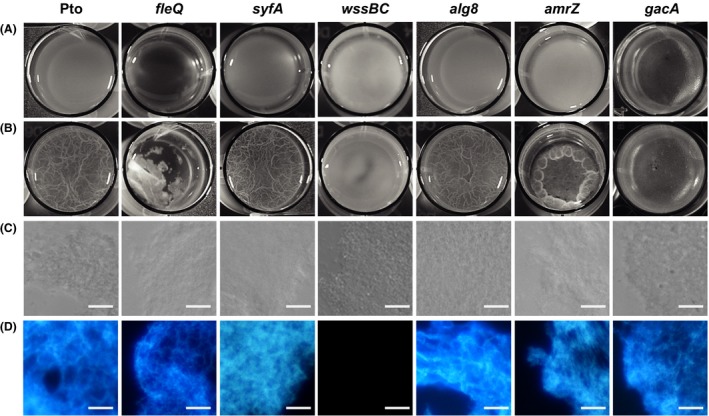
Pellicle formation by mutant strains of Pto DC3000. The different strains were grown under aerobic and static conditions at 20°C in 24 multiwell plates for 72 h in MMR supplemented with tetracycline (10 μg ml^−1^), when images were directly taken from the plate.A. Pto DC3000 strains growing in the absence of pleD* (pJB3Tc19).B. Pto DC3000 strains growing under high c‐di‐GMP intracellular levels by the presence of pleD* (pJB3pleD*). Observe that pellicles of different appearance were formed with all the strains assayed.C. Bright field microscopy images of fragments of pellicles formed in the presence of pleD*.D. Fluorescence images of the same fragments stained with calcofluor white. Note the absence of fluorescence in *wssBC* mutant. Bars = 10 μm.

The formation of pellicles under aerobic and static conditions was first studied at macroscopic level using the wild‐type strain Pto DC3000 and all the mutants mentioned above, both under physiological and high intracellular levels of c‐di‐GMP. The artificial increase of the c‐di‐GMP intracellular levels by the overexpression of PleD* in Pto DC3000 caused the formation of biofilms that are associated with the A‐L interface (i.e. floating though they are not technically buoyant), both in polystyrene multiwell plates (Fig. 1A, B and S1A) and in glass tubes (Fig. S1B). At the macroscopic level, Pto DC3000 pellicles begin their development with the formation of a thin flat film that increases its thickness over time exhibiting wrinkles on the surface. The mature biofilms have a whitish colour, are translucent and slightly adhere to the walls of the culture vessel, but readily sink if the container in which they grow is moved, suggesting strong adhesion among the cells but weak binding of the cells to the abiotic surfaces used. In the absence of pleD* (pJB3Tc19), pellicles were also formed but they were thinner, weaker, more transparent and not wrinkled (Fig S1). Also, some turbidity was observed in the liquid medium, indicating the presence of planktonic bacteria. This could point out to some difficulties in reaching the A‐L interface or deficiencies in the aggregation process, essential for the formation and maintenance of the biofilm. Furthermore, these latter pellicles were easily disaggregated with the slightest manipulation, suggesting weak adhesion among cells and weak binding to the abiotic surfaces used.

In the absence of pleD*, the formation of pellicles was observed in all the mutant strains tested; however, as in the wild type, they were thin and not wrinkled (Fig. 1A). The artificial increase of c‐di‐GMP intracellular levels favoured the formation of pellicles in aerobic and static conditions in most of the mutants tested (Fig. 1B). The *fleQ* mutant lacks the master regulator that activates the transcription of the flagellar genes in Pto DC3000, making it unable to synthesize flagella. Nonetheless, this mutant produces more syringafactin than the wild‐type strain (Nogales *et al*., 2015) and have a dual role as repressor of cellulose production and as activator in response to c‐di‐GMP (Pérez‐Mendoza *et al*., 2019). In the presence of pleD*, the pellicles of this mutant were broken and disintegrated in flocs, with a very different appearance from the wild‐type pellicles. To study the role of the biosurfactant syringafactin, we used the *syfA* mutant, which does not produce syringafactin, observing that it generated pellicles similar in appearance to those of the wild type. The mutant *wssBC*, which does not produce cellulose, also formed biofilms at the A‐L interface at high intracellular levels of c‐di‐GMP, although with a different appearance and consistency, since they were smooth and mucous, as it was previously observed (Pérez‐Mendoza *et al*., 2014). Other strains of *Pseudomonas* lacking the cellulose synthesis operon can also form biofilms, suggesting that high levels of c‐di‐GMP could activate the production of other exopolysaccharides (Friedman and Kolter, 2004; Laue *et al*., 2006; Ude *et al*., 2006; Lee *et al*., 2007; Starkey *et al*., 2009; Mann and Wozniak, 2012; Whitney and Howell, 2013). However, the Pto DC3000 *alg8* mutant, which does not produce alginate, generated pellicles of similar appearance to those of the wild type, whereas the biofilms formed by the *amrZ* mutant were much denser than those of the wild type and, at first, they floated, but in most cases they settled at the bottom of the vessels without breaking up. The *amrZ* mutant does not produce alginate, overproduces cellulose and is less motile than the wild type due to *fliC* lower expression and less flagella production (Prada‐Ramírez *et al*., 2016). In other bacterial species, GacA is involved in biofilm formation and in the regulation of exopolysaccharide production (Parkins *et al*., 2001; Yang *et al*., 2008). The Pto DC3000 *gacA* mutant produced flat and waxy‐looking pellicles, both in the absence and in the presence of PleD*, although they were slightly denser in the latter case.

In order to confirm the presence of cellulose in Pto DC300 pellicles, two types of dyes were assayed, calcofluor and calcofluor white, observing that calcofluor white specifically stained biofilm fibres (Fig. 1 and S2). When the biofilms of the different Pto DC3000 mutants (*fleQ*,* syfA*,* wssBC*,* alg8*,* amrZ* and *gacA*) were stained with the fluorochrome calcofluor white, no fluorescence was observed in the absence of pleD*. However, the wild type and all the mutants tested except *wssBC*, fluoresced in the presence of pleD*, exhibiting an extracellular matrix formed by fibres (Fig. 1D). Calcofluor stained also bacteria cells, which were clearly fluorescent in the absence of pleD* and even in the *wssBC* mutant which do not produces cellulose (Fig. S2).

### Study of pellicles by scanning electron microscopy

Scanning electron microscopy (SEM) was used to visualize in detail differences among the pellicles formed by the strains examined in this work, both in the presence and absence of pleD*. Due to the fragile consistency of Pto DC3000 pellicles, it was necessary to develop a new technique to collect and process for SEM these biofilms without damaging them. Activated carbon cloth was found to be the most suitable holder for this purpose since it allowed the adhesion of the pellicles and the SEM analysis of the face in contact with the culture medium (Fig. S3).

**Figure 2:**
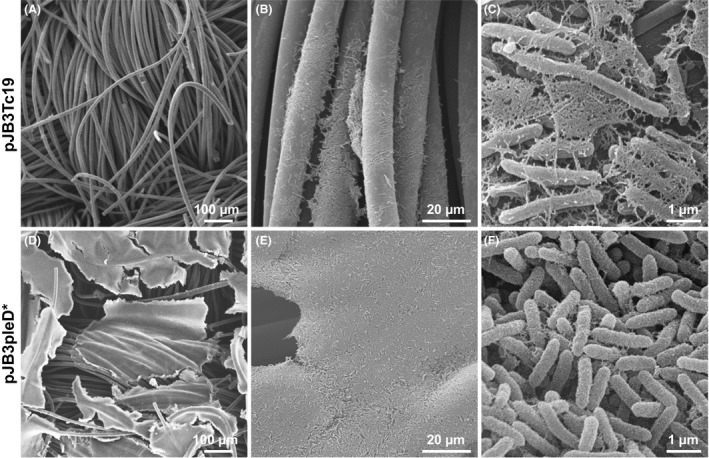
SEM images of Pto DC3000 pellicles in the absence and in the presence of pleD*. Appearance of the cells that form the Pto DC3000 pellicles at different magnifications in the absence (A, B and C) and in the presence (D, E and F) of pleD*. Bacteria were grown in MMR under aerobic and static conditions at 20°C for 72 h in multiwell plates and collected on a disc of activated carbon cloth. Note that bacteria were only visible in fracture areas of the pellicle at the highest magnification.

Pellicles formed by Pto DC3000 in the absence of pleD* appeared to be composed of very few strata of cells, very weakly bound among them. In fact, at low magnifications (100–200×), the presence of the sample on the active carbon cloth was hardly appreciated (Fig. 2A). At higher magnifications (1000–2000×), compact groups of bacteria were visible together with some scattered bacterial cells over the fibres of the activated carbon cloth (Fig. 2B). At even higher magnifications (10 000–20 000×), these cells had a uniform and smooth surface partially covered by fibres that generated an extracellular matrix that held them together (Fig. 2C).

In the presence of high c‐di‐GMP levels, the biofilms were much denser, more compact and visible even at quite low magnification (Fig. 2D and E). At higher magnifications (1000–2000×), compact pellicles were clearly visible and it was difficult to distinguish individual cells (Fig 2E). The visualization of bacterial cells in these compact pellicles was only possible at higher magnifications (10 000–20 000×) in some areas where the sample presented fractures which allowed to see bacteria with a rough surface connected by short fibres (Fig. 2F). Also, it was observed that, in the presence of pleD*, the bacteria were shorter than in its absence (Fig. 2C and F).

**Figure 3:**
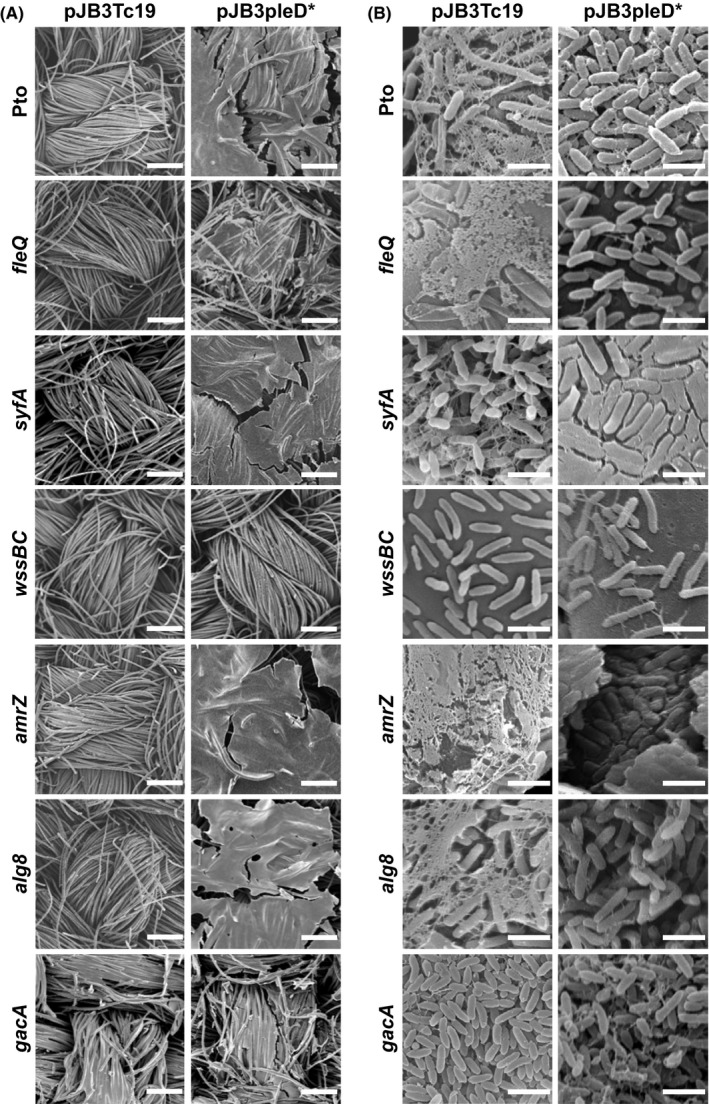
SEM images of pellicles formed by Pto and the indicated mutants both in the absence and in the presence of pleD*. Bacteria were grown in MMR under aerobic and static conditions at 20°C for 72 h in 24 multiwell plates and collected on a disc of activated carbon cloth.A. At low magnifications where cells are not visible. Bars = 100 μm.B. At higher magnifications where cells and fibres can be seen. Bars = 1 μm.

The use of activated carbon cloth as a holder for the collection and observation of pellicles has also allowed studying those generated by the mutant strains in the absence and in the presence of pleD* at SEM. Initially, low magnifications were used to find out whether differences in the overall appearance of the pellicles in the absence of pleD* could be detected, but the carbon cloth was apparently empty for all the mutants tested, as it did with the wild type (Fig. 3A). In the presence of pleD*, the pellicles of all the mutants, except *wssBC* and *gacA*, exhibited a denser and very compact appearance, similar to that of the wild type (Fig. 3A). Interestingly, no differences in the *wssBC* and *gacA* pellicles were detected between the absence and the presence of pleD*. At higher magnifications (12 000×), some differences were appreciated. In the absence of pleD*, the pellicles formed by *fleQ* were not compact and the individual cells were observed at some distance from each other, sometimes coated by a granulated material and/or by fibres similar to those observed in the wild‐type pellicles without pleD* (Fig. 3B). In the presence of pleD*, virtually no fibres were detected, the material covering the cells of this mutant in the absence of pleD* was also not present and the cells appeared a little more aggregated. In the *syfA* mutant, fibres similar to those observed with the wild type were detected, but in less amount and not covering the cells. The pellicles produced by mutants in genes related to the production of exopolysaccharides (*wssBC*,* alg8* and *amrZ*) were different. In the absence of pleD*, *wssBC* did not present fibres and the cells appeared separated, whereas in the presence of pleD* the bacterial cells were very far apart and only few cellular aggregates with some fibres similar to those of Pto DC3000 in the absence of pleD* were observed (Fig. 3B). The pellicles synthesised by the *alg8* mutant looked very similar to those of the wild type, both in the absence and in the presence of pleD* (Fig. 3). In the pellicles formed by the mutant *amrZ* in the absence of pleD*, fibres, similar to those of the wild type but much more abundant, were observed, so it was difficult to find areas of the sample where the presence of bacteria could be appreciated. In the presence of pleD*, these pellicles looked like real blocks of cells cemented to each other and covered by a dense and uniform matrix that gave them a compact and flattened appearance (Fig. 3B). As it was mentioned before, pellicles formed by the *gacA* mutant were similar in the absence and presence of pleD* and contained very few fibres. Unexpectedly, the *gacA* cells were clearly the smallest ones of all the strains studied, having a more shortened and rounded shape (Fig. 3).

### Transmission electron microscopy images of bacterial pellicles

To study the internal structure of the pellicles formed by all these strains, both in absence and in the presence of pleD*, we analysed sections by transmission electron microscopy (TEM). However, this was a very difficult task because they either disintegrated when trying to manipulate them (in the absence of pleD*), or they were bent or broken (in the presence of pleD*), thus hampering its processing for microscopy.

To obtain the samples in the absence of pleD*, the culture was decanted by means of a gentle centrifugation before they were processed for microscopy. Therefore, these preparations contained a mixture of planktonic and pellicle cells. On the other hand, biofilms produced in the presence of pleD* were fixed without centrifugation. We have developed a method to immobilize and further process both types of samples which consisted of including cell pellets and the entire biofilms in 0.8% agarose blocks, which were subsequently cut into cubes of approximately 1 mm^3^. Consequently, adding to the resin embedding procedure a pre‐embedding step in low melting agarose before dehydration enabled the successful processing of pellicle fragments without wrinkles or overlays, and obtaining cross sections of the material.

**Figure 4:**
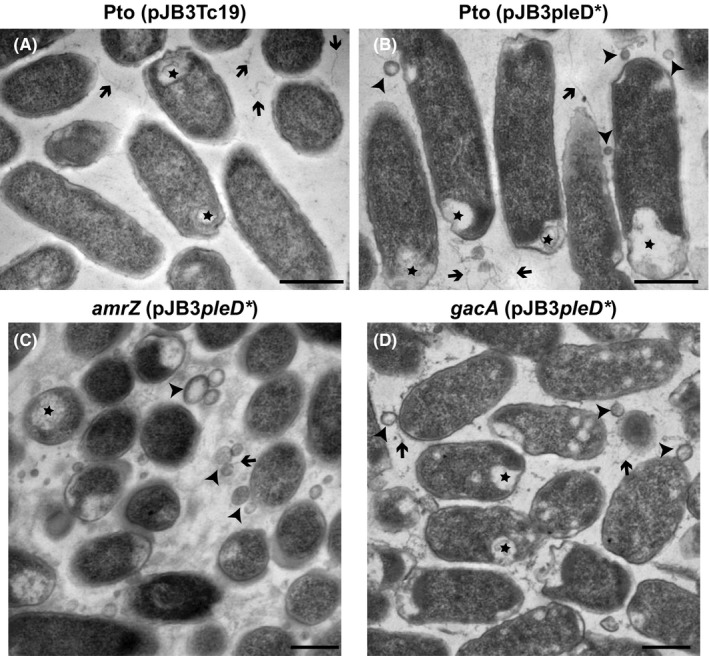
TEM images of Pto DC3000 strains in the absence and in the presence of pleD*. Ultrafine transverse sections of Pto (pJB3Tc19) cells (A), and pellicles of Pto (pJB3pleD*) (B), *amrZ* (pJB3pleD*) (C) and *gacA* (pJB3pleD*) (D). Notice the increase of fibrillar structures in the intercellular spaces (arrows), the inclusion bodies at the ends of the bacterial cells (stars), and the presence of vesicles in areas near the outer membrane of the bacterial cells (arrowheads) in pellicles formed at high c‐di‐GMP levels (in the presence of pleD*), particularly in the *amrZ* and *gacA* mutants.

The study of semithin sections by light microscopy for the analysis of the structural attributes of the Pto DC3000 pellicles was not effective for the differentiation of the individual cells and the matrix, but revealed that the thickness of the pellicles formed in the presence of pleD* was not uniform, regardless of the strain studied, and some areas have more cell layers than others (Fig S4). The use of ultrathin sections and TEM allowed obtaining a general representation of the pellicle internal structure and the cells that compose it, both in the absence and in the presence of pleD* (Fig. 4). After contrasting the ultrathin sections with uranyl acetate and lead citrate, the visualization of the samples to the TEM showed heterogeneity in the cellular distribution, since they are arranged in different positions. However, the cross sections showed that there were no significant differences between the cells in the external layers (either in contact with the air or with the medium) or those within the pellicles.

When comparing at TEM the samples in the absence and in the presence of pleD*, it was observed that, in some cases, the cells presented inclusion bodies transparent to the electrons beam that were generally located at the poles. These structures were found to be more abundant in pellicles formed in the presence of pleD* (Fig. 4). In the intercellular spaces, the presence of fibrillar structures and globular bodies of variable size was detected in both types of biofilms, being more abundant in the presence of pleD* (Fig. 4). These globular bodies had an appearance similar to the outer membrane vesicles described in other Gram‐negative bacteria, such as *P. aeruginosa* and Pto T1 (Kuehn and Kesty, 2005; Chowdhury and Jagannadham, 2013). When the pellicles of the Pto DC3000 mutants were studied, no significant differences were observed with respect to the wild type except in *gacA* and *amrZ* in the presence of pleD* (Fig. 4). In both cases, numerous globular bodies similar to the vesicles observed in the wild type were found. These bodies were especially abundant in the *amrZ* mutant.

## Discussion

The formation of biofilms is an almost universal survival strategy in bacteria that provides numerous advantages, among which we can highlight resistance to environmental stress, tolerance to antimicrobial compounds and the possibility of horizontal gene transfer (Danhorn and Fuqua, 2007). They develop on practically any biotic or abiotic solid surface in a process that generally involves three stages: adhesion of bacteria to a surface, the maturation of the biofilm and its dispersion (O'Toole *et al*., 2000; Hall‐Stoodley *et al*., 2004; Stewart and Franklin, 2008; Flemming *et al*., 2016). The adhesion of bacteria to a surface usually takes place at the solid–liquid interface; however, some bacteria are able to colonize the A‐L interface and form a floating biofilm (Branda *et al*., 2005; Armitano *et al*., 2014). Previous studies conducted in our laboratory had shown that Pto DC3000 colonizes the A‐L interface under aerobic and static conditions by forming a pellicle on the surface of the culture medium in response to the artificial increase of c‐di‐GMP levels (Pérez‐Mendoza *et al*., 2014). This pellicle takes 3 days to form, and over time, it sinks into the culture medium and disintegrates. Two hypotheses have been formulated to explain the formation of biofilms in the A‐L interface (Armitano *et al*., 2014). According to the first hypothesis, the cells would migrate to the A‐L interface, and they would first join the wall of the culture vessel to later spread on the surface. The second hypothesis proposes that aggregates of floating cells would be randomly formed on the surface acting as nuclei for the propagation of the film on the surface. The formation of floating biofilms by Pto DC3000 agrees more with the first hypothesis, since, to the minimum manipulation of the container of culture, they sink leaving a halo in the zone of the container with which they were in contact.

As shown in *Bacillus subtilis*, the formation of A‐L biofilms is a very complex process involving a wide range of genes (Angelini *et al*., 2009). Therefore, in this work, we have studied pellicles formed by Pto DC3000 using mutants in genes that could affect its formation, analysing its macroscopic aspect and structure by using different light and electron microscopy techniques. Given the fragility and thinness of these biofilms, their manipulation was very difficult, so we implemented new protocols. Thus, the TEM study of the pellicle cross sections was optimized thanks to a low melting agarose pre‐inclusion, which allowed orienting the pellicle and preventing it from bending during the processing. For the observation at the SEM, the activated carbon cloth was used, which allowed us to collect the biofilms and keep them intact until they were observed. This holder was also found to have the physical–chemical characteristics of hardness and conductivity ideal for their study at the SEM. Overall, the new methods developed in this study open up new perspectives for the analysis of fragile biofilms produced by other bacterial strains.

The activation of exopolysaccharide biosynthesis dependent on c‐di‐GMP and the subsequent formation of biofilms is a fairly widespread phenomenon in bacteria, which has also been described in different plant pathogens. In *Erwinia amylovora*, the overexpression of a DGC increases the intracellular levels of this second messenger and induces the formation of biofilms stimulating the production of cellulose, amylovoran and levan (Koczan *et al*., 2009). In *Agrobacterium tumefaciens*, the high intracellular levels of c‐di‐GMP lead to the biosynthesis of cellulose and a unipolar polysaccharide adhesin that also promote the formation of biofilms (Xu *et al*., 2013). Likewise, the deletion of the *epcB* and *epcC* genes, which encode two PDEs in *Dickeya dadantii*, increases the formation of biofilms (Yi *et al*., 2010). Increased levels of c‐di‐GMP also lead to the overexpression of partially acetylated cellulose and poly‐β‐1,6‐*N*‐acetyl‐D‐glucosamine (PGA) in the plant growth promoting and biocontrol strain *P. fluorescens* SBW25 (Spiers *et al*., 2003; Lind *et al*., 2017). The matrix of the Pto DC3000 pellicles is mainly composed of cellulose, which was initially demonstrated by its staining with calcofluor and observation of the epifluorescence LM or by quantification in liquid cultures (Pérez‐Mendoza *et al*., 2014). With the same technique but using the fluorochrome calcofluor white stain (Fluka), the presence of cellulose was identified in the biofilms of Pto DC3000 formed at high di‐c‐di‐GMP intracellular levels. The use of calcofluor white proved to be very advantageous for the clear observation of the cellulose fibres that formed the extracellular matrix of the Pto DC3000 pellicles, since with this staining agent, unlike what happened with the calcofluor, the cellulose fibres were clearly detected whilst the cells were not dyed. However, although the matrix of these biofilms is composed mainly of cellulose, this exopolysaccharide is not essential since a mutant unable to synthesize cellulose (*wssBC*) can also form pellicles, although with a very different appearance and consistency (Pérez‐Mendoza *et al*., 2014; this work). Likewise, *P. fluorescens* SBW25 is able to produce different types of A‐L biofilms with distinct phenotypes depending on the biofilm matrix components, and the wrinkly spreader biofilm, which utilizes cellulose as the primary matrix component, is the most robust and well‐attached biofilm (Koza *et al*., 2017). We should mention that Pto DC3000 lacks the *pgaABCD* operon involved in the synthesis of PGA in SBW25. On the other hand, it had previously been observed that high intracellular levels of c‐di‐GMP also induced, as in *P. aeruginosa*, the production of alginate in Pto DC3000 (Merighi *et al*., 2007; Pérez‐Mendoza *et al*., 2014; Prada‐Ramírez *et al*., 2016). However, alginate seems to be more dispensable than cellulose for the formation of these biofilms, since the *alg8* mutant, unable to synthesize this exopolysaccharide, generated pellicles indistinguishable from those produced by the wild type (Figs 1 and 2). This also explains the characteristic phenotype of the biofilms formed by the *amrZ* mutant, which does not produce alginate but overproduces cellulose (Fig. 2).

The study of the pellicles by SEM allowed the observation of the extracellular matrix details and, at the same time, revealed differences in cell morphology among the mutants. The fibres that bound the bacteria and were only distinguished in the absence of pleD*, appeared in the wild type and all the mutants except *wssBC*, which does not produce cellulose. In the presence of pleD*, the cells were immersed in a dense and uniform matrix in which it was very difficult to distinguish fibres and even bacteria. Again, the mutant *wssBC* was an exception, suggesting that the most abundant component of Pto DC3000 biofilms is cellulose.

The flagella are of great importance for the bacteria because they allow them to migrate to more favourable niches, but they also direct the initial adhesion and are involved in the maturation of the biofilm (Danhorn and Fuqua, 2007). The Pto DC3000 *fleQ* mutant, lacking the master regulator of the flagellar genes and incapable of producing flagella, formed biofilms in the presence of pleD* that disintegrated in flocs and generally deposited in the bottom of the vessel even in static conditions. The *fliC* mutant, which is unable to produce flagellin, the structural component of the flagellar filament, behaved similarly (data not shown). Therefore, regardless other effects of the *fleQ* mutation in pellicle formation, these results indicate that the flagella play an important role in the formation and maintenance of the integrity of these biofilms and agree with what is observed in *S. meliloti*, whose mutants unable to synthesize flagella have reduced ability to form biofilms (Fujishige *et al*., 2006).

The role of surfactants in the formation of bacterial biofilms is diverse, and in some species, they promote their formation whilst in others their disintegration. *P. aeruginosa* produces rhamnolipid surfactants that participate in the maintenance of the architecture of its biofilms, favouring cell–cell interactions, the union of bacteria to surfaces and the formation of channels in the extracellular matrix of the biofilm that promote the continuous flow of nutrients (Davey *et al*., 2003). *Pseudomonas* MIS38 produces a surfactant called arthrofactin that plays an important role in the development of mature biofilms since its lack results in the production of unstable and flat biofilms (Raaijmakers *et al*., 2006; Roongsawang *et al*., 2010). Likewise, in *B. subtilis* and *Staphylococcus epidermis*, surfactants promote the formation of biofilms, both at the S‐L and A‐L interfaces (Bais *et al*., 2004; Straight *et al*., 2006; Wang *et al*., 2011). On the other hand, *P. putida* PCL1445 produces putisolvin I and II, which inhibit biofilm formation not only by this bacterium but by other *Pseudomonas* strains (Kuiper *et al*., 2004). The surfactin produced by *B. subtilis* also has the ability to inhibit the formation of biofilms of various pathogenic bacteria, including *Salmonella typhimurium* or *Pseudomonas syringae* (Mireles *et al*., 2001; Bais *et al*., 2004). Pto DC3000 produces a set of linear lipopeptides called syringafactins that are essential for its swarming motility. LM and TEM revealed that, both in the absence and presence of pleD*, the *syfA* mutant produced pellicles very similar to those of the wild‐type strain. These results suggest that syringafactins do not play a role in the formation of Pto DC3000 floating biofilms. In addition, the strong inhibition of syringafactin production by high c‐di‐GMP intracellular levels (Farias, 2017) denotes the irrelevance of this surfactant in the production or maintenance of Pto DC3000 pellicles.

The Gac‐rsm is a pathway conserved in numerous Gram‐negative bacteria that allows detecting and responding to various environmental stimuli. In Pto DC3000, the GacS/GacA system activates the transcription of several small RNAs (rsmX1‐5, rsmY and rsmZ) and controls through them the expression of several alternative sigma factors involved in the response to stress, virulence and quorum sensing (Chatterjee *et al*., 2003; Vargas *et al*., 2013; Ferreiro *et al*., 2018). This pathway is essential for the synthesis of the T3SS and the polyketide coronatine, so it controls the virulence of this bacterium in tomato and the hypersensitive response in resistant plants (Chatterjee *et al*., 2003, 2007; Vargas *et al*., 2013; Ferreiro *et al*., 2018). The results obtained in this work show that the *gacA* mutant is able to develop pellicles, unlike other bacteria, such as different *Pseudomonas* or *Xylella* (Choi *et al*., 2007; Shi *et al*., 2009). As GacA is a global regulator and the Gac‐rsm pathway controls numerous processes of secondary metabolism in Pto DC3000, it is probably also involved in the detection and/or response to c‐di‐GMP levels, which would explain the similar phenotype of the biofilms of the *gacA* mutant in the absence and in the presence of pleD*.

The cross sections of the biofilms formed in the presence of pleD* and observed at the TEM showed that there were no significant morphological differences at different depths, neither between the cells in contact with the air nor with the liquid medium. On the other hand, these sections allowed the detection of inclusion bodies and extracellular vesicles, which were more abundant in the presence of pleD*. The nature of the inclusion bodies is currently under analysis. Our hypotheses are that they could be polyhydroxyalkanoate or polyglucoside granules similar to those observed in other bacteria (Shively, 1974; Bal *et al*., 1978; Jeon *et al*., 2003). The vesiculation is an important process of secretion that takes place in numerous bacteria (Toyofuku *et al*., 2019). The outer membrane vesicles (OMV) function disseminating bacterial products such as lipids, membrane proteins and other insoluble compounds. They are also involved in nutrient acquisition, cell–cell communication, stress tolerance, horizontal gene transfer, virulence and defence in human pathogenic bacteria (Grenier and Mayrand, 1987; Kuehn and Kesty, 2005; Mashburn and Whiteley, 2005; Schooling and Beveridge, 2006; Yonezawa *et al*., 2009; Chowdhury and Jagannadham, 2013; Turnbull *et al*., 2016). Despite the well‐documented function of OMVs in the interaction of bacteria with mammals, their role in phytopathogenic bacteria is largely unknown. Several studies have shed light on these structures and their possible functions in plant–bacterium interactions. The OMVs released by *Xanthomonas campestris* pv. campestris (Sidhu *et al*., 2008) contain membrane‐ and virulence‐associated proteins and are involved in the secretion of degrading enzymes of the plant cell wall (Solé *et al*., 2015). In the case of Pto T1, apart from membrane‐ and virulence‐associated proteins, they also contain the phytotoxin coronatine (Chowdhury and Jagannadham, 2013). On the other hand, OMVs produced by *Xylella fastidiosa* block its interaction with various surfaces, such as the walls of xylem vessels in host plants. Therefore, OMV production seems to be a strategy used by *X. fastidiosa* in its transition from an adhesive lifestyle, capable of insect transmission, to a motile lifestyle for systemic spread within the plant host (Ionescu *et al*., 2014). However, OMV production in pellicles formed under high c‐di‐GMP is novel features observed in Pto DC3000 that open a new pathway for the study of a different role of c‐di‐GMP in plant‐interacting bacteria.

## Experimental procedures

### Bacterial strains and growth conditions

Bacterial strains used are listed in Table 1. *P. syringae* pv. tomato DC3000 and mutants were grown statically in MMR (7 mM Na‐glutamate, 55 mM mannitol, 1.31 mM K_2_HPO_4_, 2.2 mM KH_2_PO_4_, 0.61 mM MgSO_4_, 0.34 mM CaCl_2_, 0.022 mM FeCl_3_, 0.85 mM NaCl) minimal medium (Robertsen *et al*., 1981) under aerobic and static conditions at 20°C for 72 h.

**Table 1:** Bacterial strains and plasmids used

Strain/plasmid	Relevant characteristics	References
*P. syringae* pv. tomato strains		
DC3000	Wild type; Rif^R^	Cuppels (1986)
*alg8*	*alg8::ΩSm/Sp*; Rif^R^ Sm/Sp^R^	Ferreiro *et al*. (2018)
*amrZ*	*amrZ*::Gm; Rif^R^ Gm^R^	Prada‐Ramírez *et al*. (2016)
*fleQ*	*fleQ*::ΩKm; Rif^R^ Km^R^	Vargas *et al*. (2013)
*fliC*	*fliC* (*flaA*)::miniTn5Cm; Rif^R^ Cm^R^	Hu *et al*. (2001)
*gacA* (AC811)	*gacA*::mini‐Tn5Km; Rif^R^ Km^R^	Chatterjee *et al*. (2003)
*syfA*	*ΔsyfA*; Rif^R^	Berti *et al*. (2007)
*wssBC*	*ΔwssBC*; Rif^R^	Pérez‐Mendoza *et al*. (2014)
Plasmids		
pJB3Tc19	Ap^R^, Tc^R^; cloning vector, P_lac_ promoter	Blatny *et al*. (1997)
pJB3pleD*	Ap^R^, Tc^R^; pJB3Tc19 derivate bearing a 1423 bp XbaI/EcoRI fragment containing pleD* from *C. crescentus*	Pérez‐Mendoza *et al*. (2014)

Cm^R^, Gm^R^, Km^R^, Rif^R^, Sm^R^ and Sp^R^ stand for resistance to ampicillin, chloramphenicol, gentamicin, kanamycin, rifampicin, streptomycin and spectinomycin respectively.

Plasmid pJBpleD* (Pérez‐Mendoza *et al*., 2014) was constructed by subcloning the XbaI/EcoRI fragment containing the pleD* gene from pRP89 plasmid (Paul *et al*., 2004) into the broad host range vector pJB3Tc19 (Blatny *et al*., 1997) previously digested with the same restriction enzymes. The pleD* variant carries 4 point mutations and exhibits constitutive diguanylate cyclase activity (Paul *et al*., 2004). This stably elevates the intracellular c‐di‐GMP levels independently of its regulation by phosphorylation in Pto DC3000 (Pérez‐Mendoza *et al*., 2014).

### Macroscopic appearance

The appearance of the bacterial communities at macroscopic level was studied after taking images directly from the 24 multiwell plates and glass tubes using a stereomicroscope Leica M165FC (Leica microsystems, Wetzlar, Germany).

### Cellulose detection

Calcofluor is a fluorochrome that binds non‐specifically to cellulose and chitin. Different preparations, calcofluor (Sigma‐Aldrich. St Louis, MO USA) and calcofluor white (Fluka, Sigma‐Aldrich Co., St Louis, MO, USA), were used for cellulose detection in the pellicles by fluorescence microscopy. The white stain is a mixture of calcofluor white and Evans blue. The Evans blue dye acts as a counter‐stain decreasing the background fluorescence generated by the non‐specific binding of the calcofluor, thus favouring a clearer observation of the marked structures (Harrington and Hageage, 2003).

The pellicles formed in the multiwell plate were disintegrated in 2 ml of deionized water and 8 μl of this suspension was deposited on a clean glass slide and 8 μl of calcofluor‐staining solution (5 mg ml^−1^) or 8 μl of a 1:1 mixture of 10% KOH (w/v) and calcofluor white stain were added to each sample. A coverslip was deposited on the sample that was immediately examined in an epifluorescence microscope Leica DMI600B (Leica microsystems) using UV light excitation (365 nm).

### Scanning electron microscopy

Due to the fact that it was not easy to handle bacterial pellicles without damaging them, different methods were tested, and the use of activated carbon cloth (Kynol activated carbon clothes ACC 507‐20) was chosen as the best sampling method. Carbon cloth discs of 1 cm diameter were thoroughly washed with deionized water, autoclaved (at 120°C 20 min) and then used to harvest the pellicles by touching them with the activated carbon cloth. The discs were dried on a filter paper for 15 min and fixed by immersing them in methanol for 20 min, which was later replaced with absolute ethanol, which was renewed every 30 min for three times. Bacteria were then critical point dried, sputtered with gold and observed in a SEM Jeol JSM‐6490LV (Jeol, Akishima, Japan).

### Light and transmission electron microscopy

Pellicles formed by different strains in the presence of pleD* were handled with special care in order to not disrupt them. In the absence of pleD*, pellicles were weaker, thinner and easily disintegrated when manipulating them; therefore, to obtain these samples for microscopy, the cultures were decanted by gentle centrifugation (5 min at 3000 *g*) collecting both planktonic and pellicle cells. Both types of samples were fixed in 4% paraformaldehyde and 2.5% glutaraldehyde in 0.05 M cacodylate (pH 7.2) buffer overnight at 4°C, they were then three times washed in cacodylate buffer and post‐fixed in 1% O_4_Os for 30 min at room temperature and washed again three times in the same buffer. Then, samples were pre‐embedded in 0.8% low melting agarose in water. This step was critical for the correct preservation of the pellicles. Once solidified cubes about 1 mm on each side were obtained and immediately dehydrated at 4°C in ethanol ascending series and finally embedded in Unicryl resin and polymerized for 24 h at 60 °C.

The cutting of semithin and ultrathin sections was carried out in a Reichert‐Jung Ultracut E. Semithin sections were stained with toluidine blue and examined under a Zeiss Axioskop epifluorescence microscope (Carl Zeiss, Gottingen, Germany). Ultrathin sections were stained with uranyl acetate and lead citrate and observed under a Jeol JEM‐1011 (Jeol, Akishima, Japan) transmission electron microscope.

## Conflict of interest

None declared.

## Supporting information


**Fig. S1**. Effect of overexpression of PleD* in the formation of biofilms at the air‐liquid interface.

**Fig. S2**. Fluorescence images of pellicles of Pto stained with calcofluor.

**Fig. S3**. Activated carbon cloths for collecting Pto DC3000 pellicles.

**Fig. S4**. Images of pellicles observed under the light and TEM microscope.
