# Supplementary figures and images for: Visualization and characterization of Pseudomonas syringae pv. tomato DC3000 pellicles
Source: Microb Biotechnol. 2019 Mar 5;12(4):688–702. doi: 10.1111/1751-7915.13385 (PMC6559019; doi:10.1111/1751-7915.13385)

pJB3Tc19

pJB3*pleD*\*

A

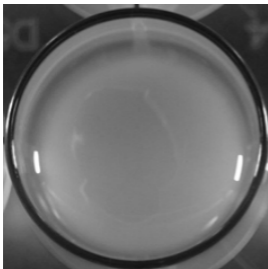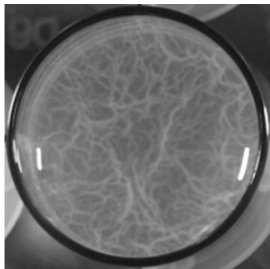

B

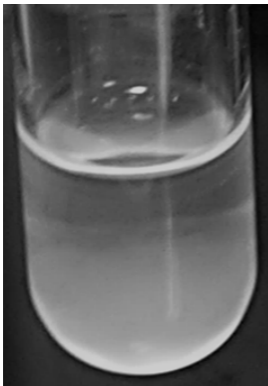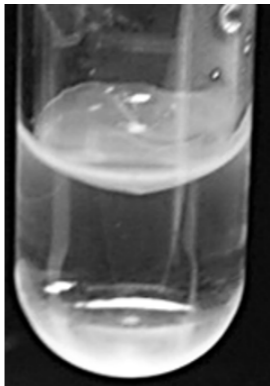

Supplement: Supplementary file 1 — Fig. S1. Effect of overexpression of PleD* in the formation of biofilms at the air‐liquid interface. [file MBT2-12-688-s001.pdf]

pJB3Tc19

pJB3pleD\*

Pto

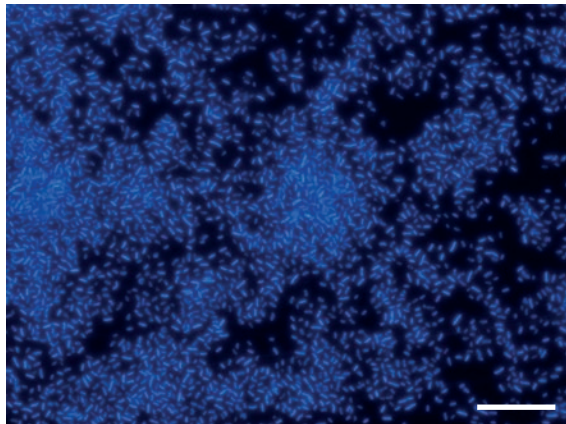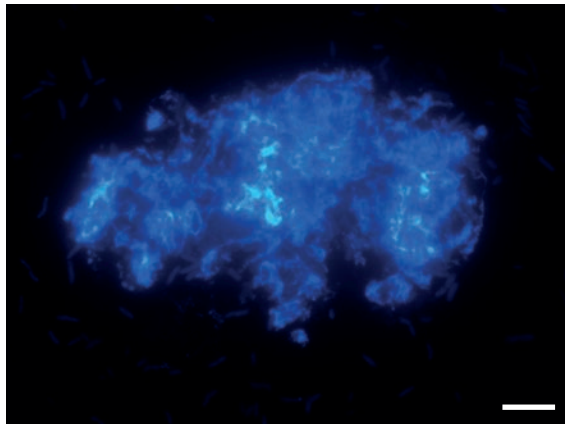

*wssBC*

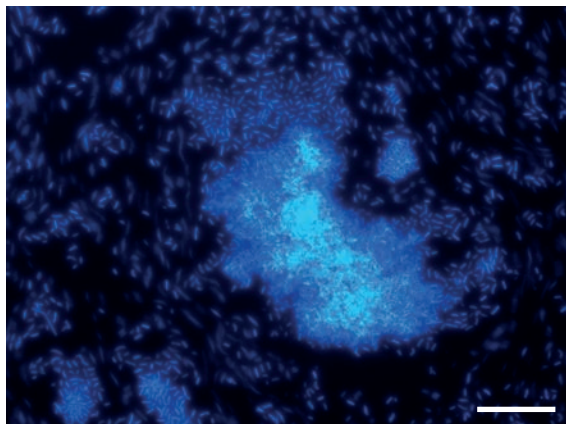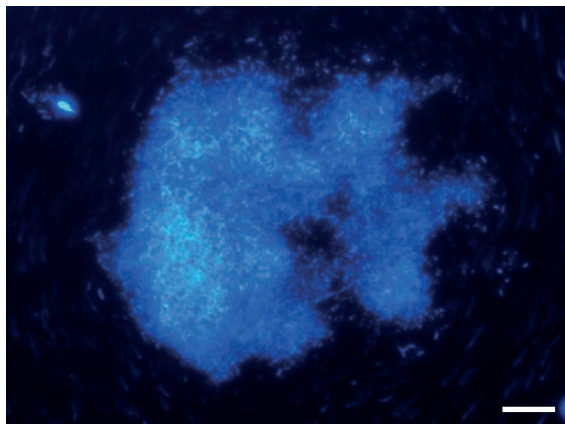



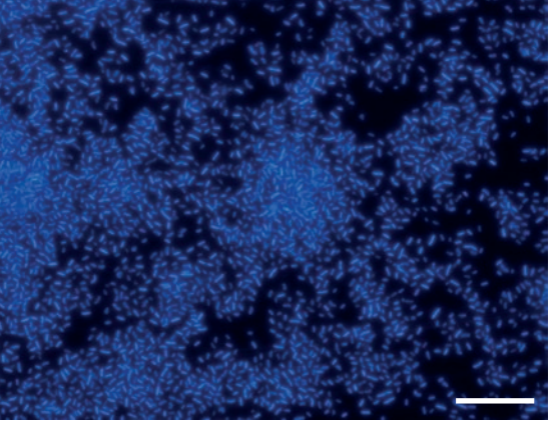

Supplement: Supplementary file 2 — Fig. S2. Fluorescence images of pellicles of Pto stained with calcofluor. [file MBT2-12-688-s002.pdf]

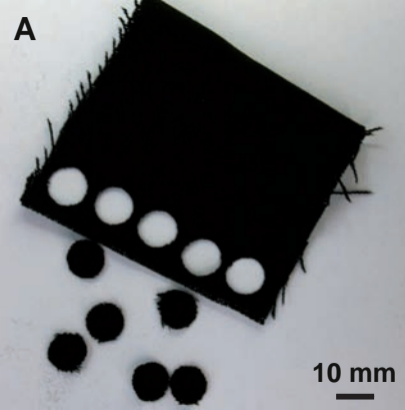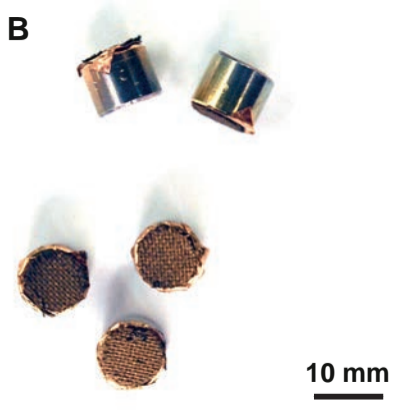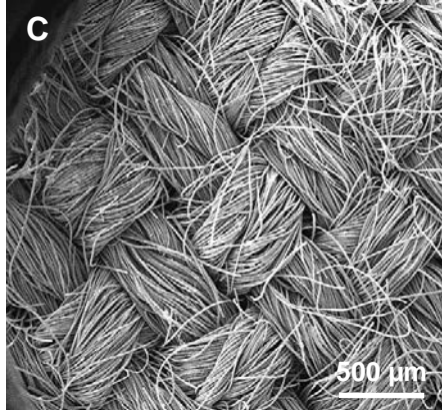

Supplement: Supplementary file 3 — Fig. S3. Activated carbon cloths for collecting Pto DC3000 pellicles. [file MBT2-12-688-s003.pdf]

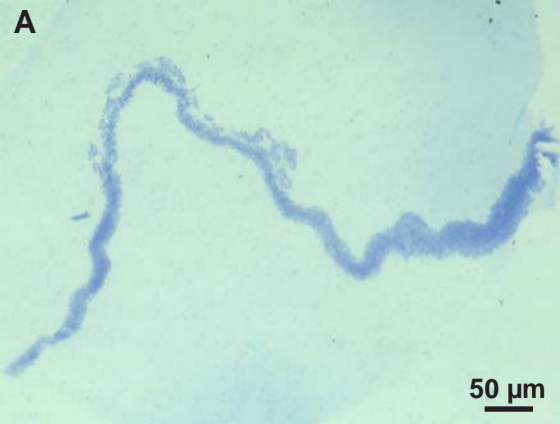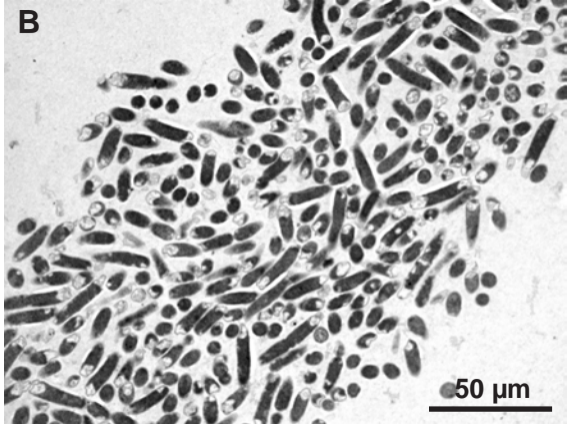

Supplement: Supplementary file 4 — Fig. S4. Images of pellicles observed under the light and TEM microscope. [file MBT2-12-688-s004.pdf]
